# Real-world characteristics & disease history of patients with X-linked hypophosphatemia before treatment with burosumab

**DOI:** 10.1007/s11657-025-01544-1

**Published:** 2025-05-13

**Authors:** Kathryn M. Dahir, Zhiyi Li, Heather M. Heerssen, Ahmed Noman, Jeremy Kim, Yang Zhao, Erik A. Imel

**Affiliations:** 1 Vanderbilt University Medical Center, Endocrinology and Diabetes, 8210 Medical Center East, 1215 21st Avenue South, Nashville, TN 37232-8148 USA; 2 Kyowa Kirin, Inc., 510 Carnegie Center Dr. Suite 600, Princeton, NJ 08540 USA; 3 Komodo Health, New York, NY USA; 4 Indiana University School of Medicine, Indianapolis, IN USA

**Keywords:** X-linked hypophosphatemia, Disease burden, Burosumab, Claims data

## Abstract

***Summary*:**

In a United States claims database, 1,358 persons with familial hypophosphatemia who began treatment with burosumab were identified. Prior to treatment, high rates of several morbidities were coded including osteoarthritis, fractures, enthesopathy, spinal stenosis, hypertension, depression and opioid use, which generally increased with age, emphasizing disease burden throughout the lifespan.

**Purpose:**

To examine the characteristics and disease history of real-world patients with X-linked hypophosphatemia (XLH) in the United States, prior to initiating burosumab.

**Methods:**

This retrospective cohort study used Komodo Health’s Healthcare Map™, a de-identified patient-level claims database. Included patients had ≥ 1 claim for familial hypophosphatemia between 01-Jan-2015 and 30-Jun-2022 (the study period) and ≥ 1 claim for burosumab between 01-Apr-2018 and 30-Jun-2022. The index date was the date of first burosumab claim. Patient demographics were measured at index; disease history was measured over the pre-index period and stratified by age. All variables were evaluated descriptively.

**Results:**

1,358 patients were included (mean age 23.5 ± 19.1 years, 847 [62%] female); 720 patients (53%) were aged < 18 years. Prior to index, patients had high levels of XLH-related morbidities. Most XLH-related morbidities appeared in the youngest age groups, and the prevalence was generally greater among the older age groups. For example, arthralgia was found in 57 patients (12%) aged ≤ 11 years and 123 patients (69%) aged ≥ 50 years. Opioid use increased with age (173 patients [24%] aged < 18 years; 328 [51%] ≥ 18 years). Physical therapy use was observed across age groups (126 patients [18%] aged < 18 years; 253 [40%] ≥ 18 years).

**Conclusions:**

At initiation of burosumab, over half of patients with XLH were < 18 years of age. Claims indicated a high prevalence of XLH-related morbidities, which began at a young age and increased over time.

**Supplementary Information:**

The online version contains supplementary material available at 10.1007/s11657-025-01544-1.

## Introduction

X-linked hypophosphatemia (XLH) is a rare genetic disorder characterized by pathological elevations in the serum concentrations of fibroblast growth factor 23 (FGF23) caused by variants of the *PHEX* gene, leading to low levels of phosphate in the blood [1, 2]. XLH usually manifests during childhood with symptoms including lower limb deformities, short stature, and dental abscesses that can persist into adulthood [2–5]. During adulthood, the clinical burden is progressive with accumulation of other complications such as fractures and pseudofractures, spinal stenosis, enthesis calcifications, hearing loss, and early onset osteoarthritis [2–5]. For example, in a study of 336 adults with XLH, over 25% of those aged 18–29 years reported having experienced a fracture, which increased to over 65% in the group aged ≥ 60 years [2].

XLH is estimated to occur in one in 20,000 to one in 60,000 individuals [3, 6–8]. The condition is associated with a significant impact on health-related quality of life, affecting both physical functioning and the ability to carry out daily activities [9]. Patients diagnosed with XLH also report negative impacts on their mental health, along with concerns for the future as the complications of the disease often progress and accumulate with age [9, 10].

Early diagnosis and treatment of XLH are important to manage disease manifestations [11]. The conventional therapy for XLH has been the active form of vitamin D (e.g., calcitriol) combined with phosphate salts [4, 11]. Although this treatment improves bone mineralization, it does not affect the renal phosphate wasting or decreased active vitamin D production that result from excessive FGF23 activity, and does not achieve persistent phosphate normalization [4, 12]. Conventional therapy is also poorly tolerated and associated with complications including nephrocalcinosis, hyperparathyroidism, and gastrointestinal distress [4, 6, 13, 14].

Burosumab, a neutralizing antibody to FGF23, first received United States (US) Food and Drug Administration approval in April 2018, and is indicated for the treatment of XLH in adult and pediatric patients aged 6 months and older [1, 15, 16]. In clinical trials, burosumab increased serum phosphate levels in both pediatric and adult patients; significantly reduced the severity of rickets in children; and improved stiffness, pain, physical functioning, and fracture/pseudofracture healing in adults [17–22]. The long-term efficacy of burosumab is maintained in both children and adults with continuous treatment, as shown by a sustained treatment effect evaluated up to 184 weeks in adults and up to 160 weeks in children, and descriptive reports finding a loss of effect upon treatment cessation in adults [23–26]. In the pivotal clinical trials and their extensions, burosumab treatment was associated with an acceptable safety profile [19, 21, 23, 27]. Over 96 weeks of treatment in adults and 160 weeks of treatment in children, most adverse events were mild to moderate in severity and no patient experienced a treatment emergent adverse event that led to treatment discontinuation or withdrawal from a study [19, 21, 23, 27].

As XLH is a rare disease and burosumab only became available to treat XLH in 2018, the clinical features and burden of disease among patients beginning treatment with burosumab have not been fully characterized. The aim of this study was to use real-world healthcare claims data to describe the demographic characteristics as well as the morbidities (XLH-related conditions), other health complications, treatments, and procedures among patients with XLH prior to starting burosumab treatment.

## Methods

### Study design

This was a retrospective study using de-identified administrative claims data from Komodo Health’s Healthcare Map™, a real-world dataset integrating disparate sources of patient-level data to map longitudinal patient journeys in the US. Data from over 150 payers are aggregated in the dataset, resulting in real-world data on more than 120 million patients who are located across all US census regions. Both open and closed healthcare claims are included in the Healthcare Map™. Open claims are those captured through practice management systems, clearinghouses, and pharmacy benefit managers [28]. They provide an overview of patient activity including items that may not have been billed, as patients may be included despite a change in insurer, and open claims are not yet adjudicated by insurers. Closed claims are those that have been submitted to an insurer, adjudicated, and paid, and thus tend to have a higher level of detail and accuracy than open claims [28]. However, the time lag is longer for closed claims and identified patient populations for a study tend to be smaller when compared with open claims because they are limited to the patient’s time within a specific insurer’s system [28]. Therefore, open claims data include more patients but may not include all claims within a certain time period, while closed claims data include a smaller number of patients but more complete claims for a given time period.

In this study, as XLH is a rare disease, combined (open and closed) claims data were used to carry out the main analysis to maximize the number of patients included in the study. This was then repeated using closed claims data only for the 1-year period prior to index as a sensitivity analysis (results shown in the Online Resources). In addition, a longitudinal subgroup analysis of patients with ≥ 5 years of continuous enrollment prior to the index date was conducted to evaluate any trends in disease history prior to burosumab treatment in both datasets.

The overall study period was 01-Jan-2015 to 30-Jun-2022. The index date was the date of the first burosumab claim. In each dataset patients were included if they had ≥ 1 claim for familial hypophosphatemia or other disorders of phosphate metabolism within the study period (International Classification of Diseases, 10th Edition, Clinical Modification [ICD-10-CM] E83.31, E83.39, ICD-9-CM 2753) and ≥ 1 claim for burosumab between 01-Apr-2018 and 30-Jun-2022. As XLH is the only form of familial hypophosphatemia for which burosumab is indicated, the combination of a familial hypophosphatemia/phosphate metabolism disorder claim and a burosumab claim was used to identify eligible patients with XLH.

### Outcomes

Patient demographics (e.g., age, sex, region, index year, payer channel) were collected on the index date. Patient morbidities and other complications were collected during the pre-index period of up to 7 years in the combined claims population and for the pre-index period of up to 1 year for the closed claims only analyses in two categories: musculoskeletal manifestations and other symptoms and conditions of interest. Treatments assessed during the pre-index period included XLH therapies and treatments (vitamin D and related agents, phosphate supplements, growth hormone) and pain medications (opioids, other prescription pain medications). Vitamin D and related agents included both the active form of vitamin D and non-active forms of vitamin D. Procedures were assessed during the pre-index period as follows: guided growth surgery; osteotomies; arthroplasty; laboratory tests (1,25-dihydroxyvitamin D; 25-hydroxyvitamin D; serum phosphate); and physical therapy.

Outcomes were assessed over the entire pre-index period and by age groups (≤ 11, 12–17, 18–29, 30–39, 40–49, ≥ 50 years). For the 5-year sub-analyses of patients with 5-year history, disease history (XLH-related morbidities, treatments, and procedures) was measured over the entire 5-year period prior to index and reported on a yearly basis, with the year furthest from index considered ‘year − 5’ and the year closest to index ‘year − 1’ (with the index date being ‘0’).

### Statistical analysis

All outcomes were evaluated descriptively using Python and R. Means, medians, standard deviations (SDs), interquartile ranges (IQRs), minimums and maximums were reported for continuous variables, and counts and proportions were reported for categorical variables.

## Results

### Sample selection

There were 2,304,549 patients with ≥ 1 claim for familial hypophosphatemia or other disorders of phosphorus metabolism between 01-Jan-2015 and 30-Jun-2022; of these, 1,358 had ≥ 1 claim for burosumab between 01-Apr-2018 and 30-Jun-2022 and were included in the combined claims population. The median time of enrollment pre-index was 1,602 days (min–max: 0–2,714 days). A total of 399 patients (29%) had at least 5 years of history were included in the 5-year sub-analyses. In addition, 643 patients met the study eligibility criteria in the closed claims only population (Online Resource Fig. 1), 56 (9%) of whom had continuous enrollment for at least 5 years and were included in the 5-year sub-analyses.

### Patient characteristics

Among the 1,358 patients in the combined claims population, the mean age was 23.5 ± 19.1 years, 62% (*n* = 847) of patients were female, and 53% (*n* = 720) were < 18 years of age. Most patients had commercial insurance (*n* = 857, 63%) and the largest proportion of patients had their first burosumab claim in 2019 (*n* = 474, 35%) (Table 1).

**Table 1:** Patient characteristics, overall and stratified by age group (combined population)

		Index age group					
Characteristics on index date	All patients *N* = 1,358	≤ 11 years *n* = 475	12–17 years *n* = 245	18–29 years *n* = 200	30–39 years *n* = 132	40–49 years *n* = 128	≥ 50 years *n* = 178
Age, mean ± SD	23.5 ± 19.1	6.4 ± 2.9	14.2 ± 1.6	22.4 ± 3.6	34.5 ± 2.9	44.5 ± 2.9	60.3 ± 7.9
Sex, *n* (%)							
Female	847 (62.4)	292 (61.5)	137 (55.9)	131 (65.5)	91 (68.9)	82 (64.1)	114 (64.0)
Index year, *n* (%)							
2018	144 (10.6)	60 (12.6)	28 (11.4)	13 (6.5)	14 (10.6)	11 (8.6)	18 (10.1)
2019	474 (34.9)	167 (35.2)	104 (42.4)	74 (37.0)	39 (29.5)	34 (26.6)	56 (31.5)
2020	346 (25.5)	118 (24.8)	51 (20.8)	56 (28.0)	30 (22.7)	34 (26.6)	57 (32.0)
2021	287 (21.1)	100 (21.1)	46 (18.8)	36 (18.0)	33 (25.0)	38 (29.7)	34 (19.1)
2022	107 (7.9)	30 (6.3)	16 (6.5)	21 (10.5)	16 (12.1)	11 (8.6)	13 (7.3)
Region, *n* (%)							
Northeast	220 (16.2)	66 (13.9)	41 (16.7)	35 (17.5)	27 (20.5)	16 (12.5)	35 (19.7)
Midwest	365 (26.9)	133 (28.0)	59 (24.1)	51 (25.5)	35 (26.5)	34 (26.6)	53 (29.8)
South	553 (40.7)	218 (45.9)	103 (42.0)	75 (37.5)	44 (33.3)	61 (47.7)	52 (29.2)
West	220 (16.2)	58 (12.2)	42 (17.1)	39 (19.5)	26 (19.7)	17 (13.3)	38 (21.3)
Payer channel, *n* (%)							
Commercial	857 (63.1)	237 (49.9)	139 (56.7)	147 (73.5)	100 (75.8)	101 (78.9)	133 (74.7)
Managed Medicaid/Medicaid	450 (33.1)	238 (50.1)	106 (43.2)	50 (25.0)	26 (19.7)	21 (16.4)	9 (5.1)
Medicare	14 (1.0)	0 (0.0)	0 (0.0)	0 (0.0)	2 (1.5)	1 (0.8)	11 (6.2)
Medicare Advantage	37 (2.7)	0 (0.0)	0 (0.0)	3 (1.5)	4 (3.0)	5 (3.9)	25 (14.0)

*IQR* interquartile range, *SD* standard deviation, *XLH* X-linked hypophosphatemia

Thirty-five percent of patients (*n* = 475) were in the ≤ 11 years age group, 18% (*n* = 245) were in the 12–17 years age group, 15% (*n* = 200) were in the 18–29 years age group, 10% (*n* = 132) were in the 30–39 years age group, 9% (*n* = 128) were in the 40–49 years age group, and 13% (*n* = 178) were in the ≥ 50 years age group. The majority of patients were female across all age groups. Most patients had commercial insurance across age groups, although the proportion was greater in older patients (50% [*n* = 237] of patients aged ≤ 11 years vs. 75% [*n* = 133] aged ≥ 50 years). Similar characteristics were seen in the closed claims population, with a slight difference in insurance coverage – in younger age groups, more patients had managed Medicaid/Medicare insurance (Online Resource Table 1).

### Morbidities and other complications

**Fig. 1:**
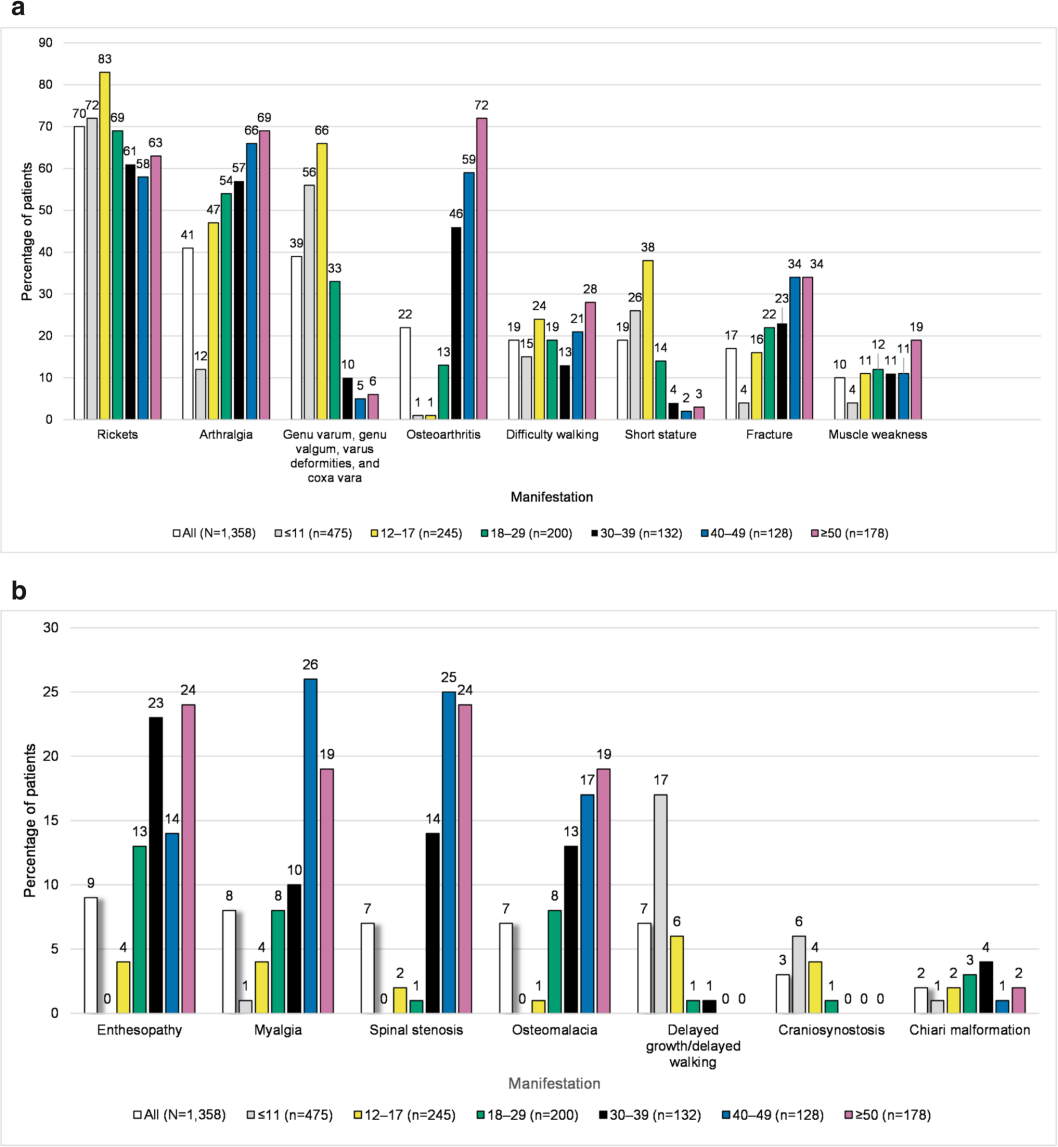
**a** Musculoskeletal manifestations by age group (combined population) (Fig. 1a). The bar chart depicts musculoskeletal manifestations determined by diagnosis coding, by age group in the combined population. The Y-axis is the percentage of patients, and the X-axis is manifestations, with each manifestation having a bar for the overall population and a separate bar for each age category. The number of patients in each category is: overall 1,358; ≤ 11 years 475; 12–17 years 245; 18–29 years 200; 30–39 years 132; 40–49 years 128; and ≥ 50 years 178. The percentage of patients with each manifestation, listed in the order they appear from left to right (starting with the overall population, and continuing from youngest to oldest age group) is: rickets (70, 72, 83, 69, 61, 58, 63); arthralgia (41, 12, 47, 54, 57, 66, 69); genu varum, genu valgum, varus deformities, and coxa vara (39, 56, 66, 33, 10, 5, 6); osteoarthritis (22, 1, 1, 13, 46, 59, 72); difficulty walking (19, 15, 24, 19, 13, 21, 28); short stature (19, 26, 38, 14, 4, 2, 3); fracture (17, 4, 16, 22, 23, 34, 34); muscle weakness (10, 4, 11, 12, 11, 11, 19). **b **Additional musculoskeletal manifestations by age group (combined population) (Fig. 1b). The bar chart depicts additional musculoskeletal manifestations by age group in the combined population. The Y-axis is the percentage of patients, and the X-axis is manifestations, with each manifestation having a bar for the overall population and a separate bar for each age category. The number of patients in each category is: overall 1,358; ≤ 11 years 475; 12–17 years 245; 18–29 years 200; 30–39 years 132; 40–49 years 128; and ≥ 50 years 178. The percentage of patients with each manifestation, listed in the order they appear from left to right (starting with the overall population, and continuing from youngest to oldest age group) is: enthesopathy (9, 0, 4, 13, 23, 14, 24); myalgia (8, 1, 4, 8, 10, 26, 19); spinal stenosis (7, 0, 2, 1, 14, 25, 24); osteomalacia (7, 0, 1, 8, 13, 17, 19); delayed growth/delayed walking (7, 17, 6, 1, 1, 0, 0); craniosynostosis (3, 6, 4, 1, 0, 0, 0); chiari malformation (2, 1, 2, 3, 4, 1, 2)

The most common musculoskeletal conditions in the combined claims population were rickets (70% [*n* = 951]); arthralgia (41% [*n* = 563]); genu varum, genu valgum, varus deformities, and/or coxa vara (39% [*n* = 524]); osteoarthritis (22% [*n* = 297]), and difficulty walking (19% [*n* = 262]) (Fig. 1a, b). Rates of musculoskeletal conditions were generally lower in the closed population, reflecting the shorter period over which claims were captured (1 year vs 7 years for the combined populations) (Online Resource Fig. 2a and 2b).

**Fig. 2:**
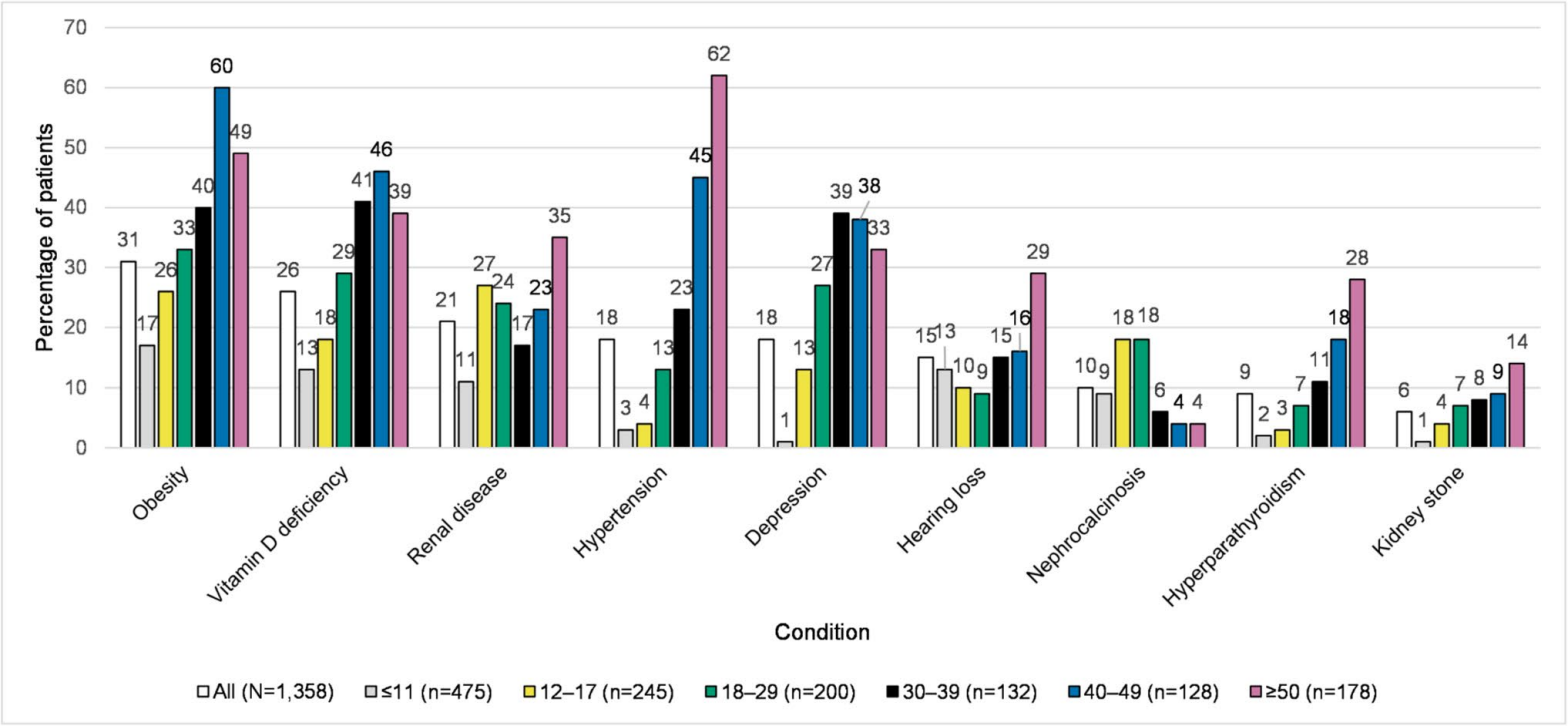
Other symptoms and conditions of interest by age group (combined population) (Fig. 2). The bar chart depicts other symptoms and conditions of interest by age group in the combined population. The Y-axis is the percentage of patients, and the X-axis is symptoms or conditions of interest, with each of these having a bar for the overall population and a separate bar for each age category. The number of patients in each category is: overall 1,358; ≤ 11 years 475; 12–17 years 245; 18–29 years 200; 30–39 years 132; 40–49 years 128; and ≥ 50 years 178. The percentage of patients with each symptom or condition of interest, listed in the order they appear from left to right (starting with the overall population, and continuing from youngest to oldest age group) is: obesity (31, 17, 26, 33, 40, 60, 49); vitamin D deficiency (26, 13, 18, 29, 41, 46, 39); renal disease (21, 11, 27, 24, 17, 23, 35); hypertension (18, 3, 4, 13, 23, 45, 62); depression (18, 1, 13, 27, 39, 38, 33); hearing loss (15, 13, 10, 9, 15, 16, 29); nephrocalcinosis (10, 9, 18, 18, 6, 4, 4); hyperparathyroidism (9, 2, 3, 7, 11, 18, 28); kidney stone (6, 1, 4, 7, 8, 9, 14)

Claims for obesity were noted in 31% (*n* = 426) of patients in the combined population, vitamin D deficiency in 26% (*n* = 349), renal disease in 21% (*n* = 280), hypertension in 18% (*n* = 249), depression in 18% (*n* = 247), and fracture in 17% (*n* = 232), with 15% (*n* = 197) having claims associated with codes for hearing loss (Fig. 2). Lower rates of these symptoms and conditions of interest were also observed in the closed population (Online Resource Fig. 3).

**Fig. 3:**
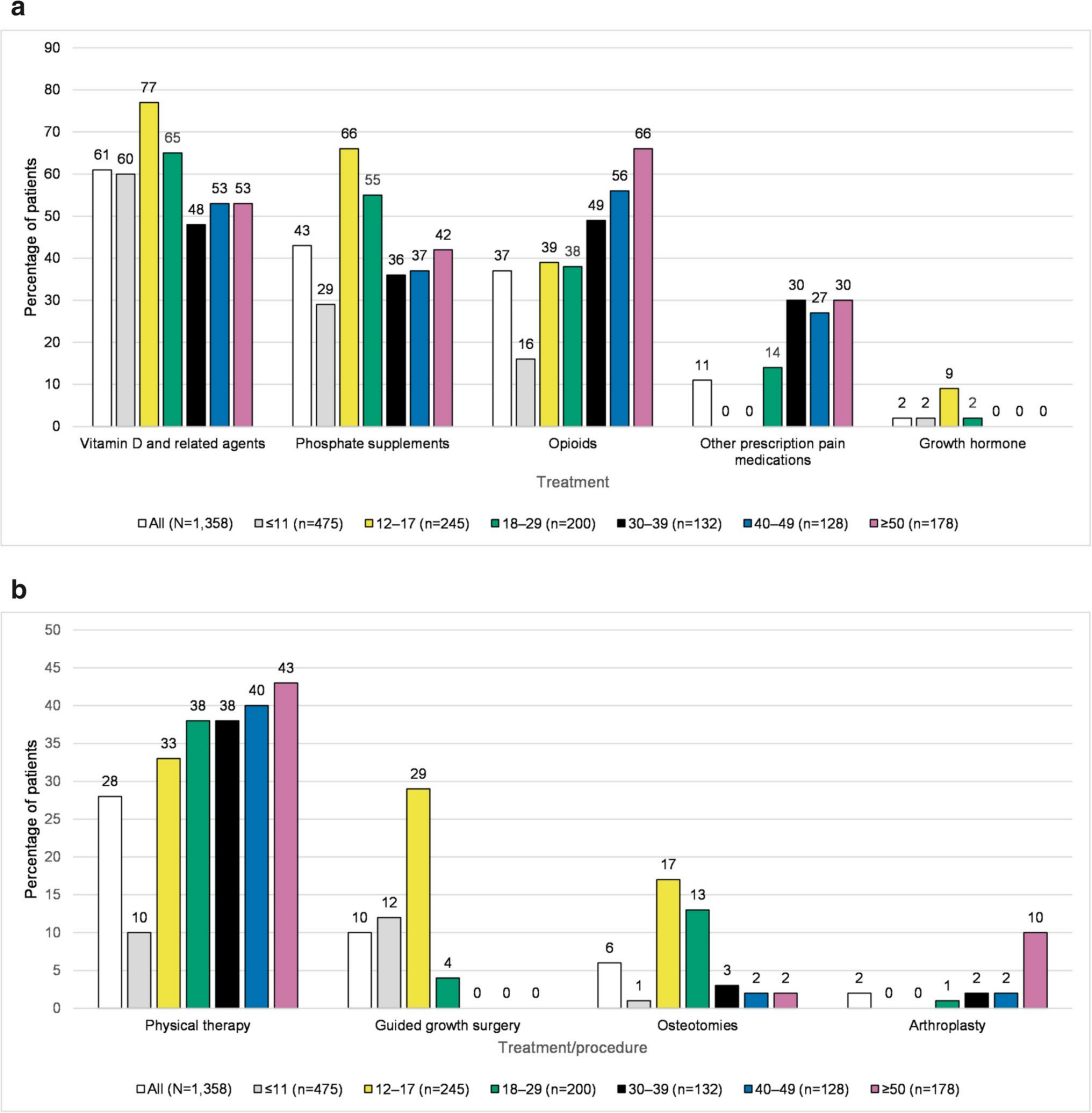
**a** Medical treatments by age group (combined population) (Fig. 3a). The bars depict medical treatments by age group in the combined population. The Y-axis is the percentage of patients, and the X-axis is medical treatments, with each of these having a bar for the overall population and a separate bar for each age category. The number of patients in each category is: overall 1,358; ≤ 11 years 475; 12–17 years 245; 18–29 years 200; 30–39 years 132; 40–49 years 128; and ≥ 50 years 178. The percentage of patients with each medical treatment, listed in the order they appear from left to right (starting with the overall population, and continuing from youngest to oldest age group) is: vitamin D and related agents (61, 60, 77, 65, 48, 53, 53); phosphate supplements (43, 29, 66, 55, 36, 37, 42); opioids (37, 16, 39, 38, 49, 56, 66); other prescription pain medications (11, 0, 0, 14, 30, 27, 30); growth hormone (2, 2, 9, 2, 0, 0, 0). **b** Other treatments and procedures (combined population) (Fig. 3b). The bar chart depicts other treatments and procedures by age group in the combined population. The Y-axis is the percentage of patients, and the X-axis is treatments and procedures, with each of these having a bar for the overall population and a separate bar for each age category. The number of patients in each category is: overall 1,358; ≤ 11 years 475; 12–17 years 245; 18–29 years 200; 30–39 years 132; 40–49 years 128; and ≥ 50 years 178. The percentage of patients with each treatment or procedure, listed in the order they appear from left to right (starting with the overall population, and continuing from youngest to oldest age group) is: physical therapy (28, 10, 33, 38, 38, 40, 43); guided growth surgery (10, 12, 29, 4, 0, 0, 0); osteotomies (6, 1, 17, 13, 3, 2, 2); arthroplasty (2, 0, 0, 1, 2, 2, 10)

When examined by age group, higher proportions of patients in older age groups than younger groups experienced most musculoskeletal and other symptoms and conditions of interest in the combined population (Fig. 1a, Fig. 1b, Fig. 2). For example, arthralgia claims occurred in 12% (*n* = 57) of the ≤ 11 years age group and 69% (*n* = 123) of the ≥ 50 years age group. Osteoarthritis claims were present in 72% (*n* = 129) of patients aged ≥ 50 years, and only 2% (*n* = 6) of those < 18 years. Vitamin D deficiency claims were present in only 13% (*n* = 63) of those aged ≤ 11 years and 39% (*n* = 70) of patients aged ≥ 50 years. Renal disease claims were present in 11% (*n* = 51) of the ≤ 11 years age group and 35% (*n* = 63) of the ≥ 50 years age group. Hypertension claims were present in 3% (*n* = 14) of the ≤ 11 years age group and 62% (*n* = 111) of the ≥ 50 years age group. Claims for fracture occurred in 4% (*n* = 18) of those in the ≤ 11 years age group and 34% (*n* = 60) of the ≥ 50 years age group. Although few patients overall had a code for kidney stones or hyperparathyroidism, these were mainly in older years. In contrast, nephrocalcinosis was primarily diagnosed in patients < 30 years old. Other conditions were also more prevalent in the younger population, such as craniosynostosis, for which claims occurred in 6% (*n* = 30) of those aged ≤ 11 years and none of those aged ≥ 30 years. As expected, claims for delayed growth/delayed walking also occurred in children as a pediatric manifestation, with 13% (*n* = 96) of those < 18 years old having a claim vs no patients aged ≥ 40 years having a claim. These age group results were largely confirmed in the closed claims population (Online Resource Fig. 2a, Fig. 2b, Fig. 3).

### Medications and procedures

The majority of patients had received a therapy for XLH, with 61% (*n* = 829) receiving vitamin D and related agents and 43% (*n* = 581) receiving phosphate supplementation (Fig. 3a). Use of supplements was highest in the 12–17 years age group. In this group, 77% (*n* = 188) had a claim for vitamin D and related agents and 66% (*n* = 162) of patients had a claim for phosphate supplements. In comparison, in the ≥ 50 years age group, 42% (*n* = 75) of patients had a claim for phosphate supplements and 53% (*n* = 94) a claim for vitamin D and related agents. Growth hormone was prescribed to 2% (*n* = 31) of patients, 90% (*n* = 28) of whom were < 18 years of age, and 10% (*n* = 3) of whom were in the 18–29 years age group (Fig. 3a). Use of pain medication was more common in the older age groups than younger; opioids were used by 16% (*n* = 78) of those aged ≤ 11 years compared with 66% (*n* = 117) of those aged ≥ 50 years, and other prescription pain medications were used by less than 1% (*n* = 1) of those aged ≤ 11 years compared with 30% (*n* = 53) of those aged ≥ 50 years. Although overall rates of medication usage were lower in the closed population, the age group trends were consistent with those observed in the combined population (Online Resource Fig. 4a).

During the pre-index period, 76% (*n* = 1,030) of patients underwent a laboratory test for serum phosphate and 70% (*n* = 953) had a test for 25-hydroxyvitamin D. A similar proportion of patients received laboratory tests across age groups. Overall, 28% (*n* = 379) of patients received physical therapy, and more older patients received physical therapy than younger patients (43% [*n* = 76] in the ≥ 50 years age group, 10% [*n* = 46] in the ≤ 11 years age group) (Fig. 3b). Overall, 6% (*n* = 84) of patients received osteotomies during the study period, with the majority occurring in the 12–17 (17% [*n* = 42]) and 18–29 (13% [*n* = 26]) age groups. Age group results for treatments and procedures were similar in the closed population (Online Resource Fig. 4b).

### 5-year sub-analyses

Among the 399 adults with at least 5 years of medical and pharmacy claims history in the combined claims population, the mean age was 41.2 ± 16.5 years (Table 2). The prevalence of most musculoskeletal manifestations remained relatively steady over 5 years (Table 3). Osteoarthritis, however, increased throughout the 5 years, from 18% (*n* = 73)] in year − 5 to 29% (*n* = 114)] in year − 1. Arthralgia increased slightly from 24% (*n* = 97) in year − 5 to 32% (*n* = 128) in year − 1. Among other symptoms and conditions of interest, the prevalence of obesity increased by ≥ 10 percentage points between year − 5 (14%; *n* = 73) and year − 1 (27%; *n* = 109). The prevalence of most medications and procedures remained steady over 5 years, although some increased over time. Opioids were used by 23% (*n* = 90) of patients in year − 5 and 28% (*n* = 111) in year − 1, and other prescription pain medications were used by 6% (*n* = 22) of patients in year − 5 and 12% (*n* = 47) in year − 1. Laboratory tests such as 25-hydroxyvitamin D (25% [*n* = 100] in year − 5 to 55% [*n* = 220] in year − 1) and serum phosphate (25% [*n* = 99] in year − 5 to 60% [*n* = 241] in year − 1) showed increasing utilization over time. Physical therapy showed a slight increase in use throughout the years (11% [*n* = 44] in year − 5 to 17% [*n* = 68] in year − 1). Results for the 5-year sub-analyses were generally similar in the closed claims only population, with higher proportions of laboratory tests in the closed claims cohort in some years (Online Resource Table 2, Online Resource Table 3).

**Table 2:** Patient characteristics in the 5-year subgroup (combined population)

	All patients
	*n* = 399
Characteristics on the index date	
Age, mean ± SD	41.2 ± 16.5
Age group, n (%)	
18–29	118 (29.6)
30–39	76 (19.0)
40–49	76 (19.0)
≥ 50	129 (32.3)
Sex, n (%)	
Female	269 (67.4)
Index year, n (%)	
2019	125 (31.3)
2020	125 (31.3)
2021	107 (26.8)
2022	42 (10.5)
Region, n (%)	
Northeast	78 (19.5)
Midwest	116 (29.1)
South	131 (32.8)
West	74 (18.5)
Payer channel, n (%)	
Commercial	297 (74.4)
Managed Medicaid/Medicaid	66 (16.5)
Medicare	11 (2.8)
Medicare Advantage	25 (6.3)

**Table 3:** Morbidities and other complications in the 5-year population (combined population)

	Years prior to index date				
	Year − 5	Year − 4	Year − 3	Year − 2	Year − 1
	*n* = 399	*n* = 399	*n* = 399	*n* = 399	*n* = 399
Musculoskeletal manifestations, *n* (%)					
Rickets	135 (33.8)	134 (33.6)	109 (27.3)	125 (31.3)	126 (31.6)
Arthralgia	97 (24.3)	123 (30.8)	136 (34.1)	121 (30.3)	128 (32.1)
Genu varum, genu valgum, varus deformities, and coxa vara	30 (7.5)	31 (7.8)	21 (5.3)	21 (5.3)	27 (6.8)
Osteoarthritis	73 (18.3)	94 (23.6)	108 (27.1)	104 (26.1)	114 (28.6)
Difficulty walking	27 (6.8)	30 (7.5)	32 (8.0)	36 (9.0)	37 (9.3)
Short stature	11 (2.8)	6 (1.5)	8 (2.0)	8 (2.0)	13 (3.3)
Fracture	42 (10.5)	50 (12.5)	43 (10.8)	52 (13.0)	45 (11.3)
Muscle weakness	16 (4.0)	17 (4.3)	23 (5.8)	19 (4.8)	18 (4.5)
Enthesopathy	14 (3.5)	27 (6.8)	28 (7.0)	24 (6.0)	31 (7.8)
Myalgia	9 (2.3)	19 (4.8)	25 (6.3)	29 (7.3)	27 (6.8)
Spinal stenosis	9 (2.3)	13 (3.3)	28 (7.0)	22 (5.5)	34 (8.5)
Osteomalacia	9 (2.3)	14 (3.5)	26 (6.5)	31 (7.8)	35 (8.8)
Delayed growth/delayed walking	0 (0.0)	0 (0.0)	1 (0.3)	0 (0.0)	2 (0.5)
Craniosynostosis	1 (0.3)	0 (0.0)	0 (0.0)	1 (0.3)	0 (0.0)
Chiari malformation	1 (0.3)	3 (0.8)	5 (1.3)	8 (2.0)	7 (1.8)
Other symptoms and conditions of interest, *n* (%)					
Obesity	57 (14.3)	75 (18.8)	97 (24.3)	105 (26.3)	109 (27.3)
Vitamin D deficiency	49 (12.3)	53 (13.3)	57 (14.3)	76 (19.1)	87 (21.8)
Renal disease	35 (8.8)	54 (13.5)	54 (13.5)	57 (14.3)	52 (13.0)
Hypertension	78 (19.6)	98 (24.6)	110 (27.6)	120 (30.1)	113 (28.3)
Depression	57 (14.3)	64 (16.0)	78 (19.6)	73 (18.3)	92 (23.1)
Hearing loss	16 (4.0)	24 (6.0)	29 (7.3)	35 (8.8)	22 (5.5)
Nephrocalcinosis	8 (2.0)	14 (3.5)	12 (3.0)	17 (4.3)	17 (4.3)
Hyperparathyroidism	18 (4.5)	28 (7.0)	26 (6.5)	44 (11.0)	39 (9.8)
Kidney stone	8 (2.0)	19 (4.8)	12 (3.0)	16 (4.0)	13 (3.3)

## Discussion

This study utilized healthcare insurance claims data to further understand the impact of XLH on patients in terms of morbidities and other complications, as well as medications and procedures. The results suggest that prior to receiving burosumab, patients with XLH experienced a substantial and accumulating disease burden. In the study, analyses were carried out in a cohort containing both open and closed (combined) claims data; these analyses were then repeated using closed claims only. As the closed claims results largely confirmed the combined claims results, we will discuss only the combined results in detail here.

This study reaffirmed previous findings on the substantial disease burden associated with XLH [2, 5, 29]. However, the rates of some clinical manifestations observed in this study were lower than seen in previous studies, possibly due to variations in data sources, time periods covered, lack of diagnostic coding for the conditions, and lack of inclusion of uninsured patients [2, 5, 29]. For example, while previous research reports enthesopathy rates of 27–99% in patients with XLH [5, 20], our study found relatively few individuals (9% [*n* = 128]) had claims for this condition. Similarly, the nephrocalcinosis rates were far lower (10% [*n* = 139]) than the reported ~ 50% in previous XLH-related publications [30, 31]. Both of these suggest clinical undercoding or underdiagnosis for these medically important conditions. In our study, manifestations of XLH were identified based on diagnosis and procedural codes within a period of ≤ 7 years prior to burosumab treatment, whereas other studies relied on self-reported outcomes from any time in a patient’s cumulative lifetime disease history, or on direct assessment using testing conducted in a research setting [5, 20]. Thus, occurrences over a ≤ 7-year period may underestimate the lifetime burden. Furthermore, some conditions (such as enthesopathy and nephrocalcinosis) reported at a higher prevalence in studies at tertiary expert centers may not be regularly screened or coded for in insurance claims. Patients in this study had a relatively low occurrence of procedures compared with rates in the existing literature. For example, a previous study reported that 71% (*n* = 70/99) of surveyed adult patients had undergone osteotomy [32]. In the present study, only 6% (*n* = 94) of patients had osteotomies during the study period. This difference is likely attributable to the study design measuring only occurrences during a discrete period of observation, which could not account for procedures occurring over the entire preceding lifetime, such as those undergone in childhood by patients who were adults during the study period.

Although several studies have examined the impact of XLH, few have assessed the accumulative nature of the disease in relation to advancing age [2]. The chronic nature of phosphate wasting in XLH can lead to long-term sequelae involving multiple organ systems [33]. Previous research has shown that adults with XLH endure ongoing clinical manifestations such as skeletal deformities, fractures and pseudofractures, pain, dental abnormalities, and impaired physical function and mobility, which in the aggregate may diminish their quality of life and restrict daily activities [29, 34]. The cumulative disease burden of XLH in adults can be profound and multifaceted, reflecting significant unmet needs [29, 33]. In addition, adults with XLH face substantial socioeconomic burdens due to disability and high healthcare resource utilization [29]. Our study using claims data adds to prior research on the accumulative burden of XLH by demonstrating the prevalence of manifestations in defined age groups across the lifetime of patients, and is consistent with the results of Javaid et al.’s analysis of data from clinical trial and direct patient survey populations [2]. Overall, the results suggest that greater awareness and understanding of XLH’s lifelong impact are crucial for improving care strategies, ensuring effective management, and optimizing patient outcomes through a multidisciplinary approach that bridges pediatric and adult healthcare services.

When we assessed the prevalence of clinical manifestations of XLH and other morbidities in patients stratified by age, upward trends were found across advancing age groups. Musculoskeletal and other symptoms and conditions of interest, such as osteoarthritis, arthralgia, fractures, hypertension, and renal disease, were more prevalent in older vs younger age groups. However, it is notable that many conditions that are typically experienced by older individuals, such as osteoarthritis, were observed in young adults with XLH, indicating early onset of morbidities in this population, similar to Javaid et al. [2]. In addition, certain musculoskeletal manifestations, including craniosynostosis, short stature, and delayed walking, were identified primarily in younger age groups, because these conditions are typically managed in children rather than adults. Nonetheless, however, these conditions can have lifelong implications.

Non-musculoskeletal manifestations were also relatively common in this study population. Notably, depression was experienced relatively frequently. Overall, 18% (*n* = 247) of patients had a claim for this condition during the study period, including 39% (*n* = 52) in the 30–39 years age group. In comparison, 18% of adults have experienced depression in their lifetime in the US population as a whole [35], indicating that the burden of XLH may extend beyond the known physical impacts of the disease. These results suggest that healthcare practitioners should consider screening patients with XLH for depression. In addition, components of metabolic syndrome were frequent in the study population and escalated in adulthood. For example, about 40% (*n* = 53) of patients in their thirties were diagnosed with obesity compared with 60% (*n* = 77) of those in their forties, and corresponding rates of hypertension were 23% (*n* = 31) and 45% (*n* = 57).

Use of pain medications was common in the evaluated patient data. Overall, 37% (*n* = 501) of patients were prescribed opioids, and 11% (*n* = 155) were prescribed other pain medications. Both opioids and other pain medications were increasingly used with advancing age. This increase was statistically significant in the closed population (Online Resource Fig. 4a). While opioid use was generally higher in patients aged 18 years and above, use was also documented in almost a quarter of children (173 [24%] patients aged < 18 years). This may have been due to brief opioid use in conjunction with surgery and/or other procedures, although our available data could not address this question. Nonetheless, this finding warrants further research into opioid use among children with XLH. Importantly, rates of non-opioid pain medication use were likely underestimated as the study could not capture over-the-counter treatments. Future studies should evaluate chronic use of pain medication in patients with XLH, as this study did not elucidate the nature of opioid use beyond whether opioids were prescribed on any occasion.

By assessing a subgroup of our adult patients longitudinally over a 5-year period (*n* = 399) prior to their first burosumab claim, we were able to further demonstrate the accumulative nature of the disease complications over time. In these analyses, the incidence of coding for most morbidities either persisted or, in some cases, increased throughout the 5-year period. Specifically, while there was some fluctuation in codes for osteoarthritis, myalgia, spinal stenosis, and enthesopathy, overall, they showed a gradual increase in frequency over the 5-year period.

In some cases, unexpected claims were observed in the adult population. For example, claims for rickets were documented among adult patients in this study. Rickets is a pediatric condition that is no longer present in adults with closed growth plates. However, the sequelae of rickets remain, as does the underlying biochemical pathophysiology in adults with XLH. Thus, it is unclear whether the presence of this code represents misdiagnosis in this population or, more likely, the misapplication of a pediatric code based on their past history and ongoing hypophosphatemia. Patients may have been provided with a rickets code when a code for osteomalacia would have been more appropriate. Alternatively, a childhood code of rickets may persist into adulthood due to a patient’s past history of rickets, or because XLH is often referred to as ‘X-linked hypophosphatemic rickets’. In our clinical experience, adult patients with XLH often still refer to their condition with inclusion of the term “rickets”. Three patients (1.5%) in the 18–29 years age group had a claim for growth hormone, which is unexpected since this treatment should be prescribed to individuals with open growth plates (i.e., younger than 18 years). It is possible these patients were closer to the younger end of the age range when this claim was made, or may have had a separate diagnosis of growth hormone deficiency.

This study has several strengths, including the use of both combined claims data and closed claims data alone. Administrative claims data contain information on diagnoses, procedures, treatments, source and setting of care, date of service, and demographic information for large, diverse patient populations allowing for more sophisticated patient analyses. The large sample size also allows for a more comprehensive picture of patient populations, particularly with rare diseases, which improves the generalizability of the study. In addition, this study examined XLH morbidities, treatments, and procedures both stratified by age group and over a span of 5 years in a subgroup of patients, providing insight into the progressive nature of the disease over time.

This study also has some limitations, mainly related to the data sources utilized. Claims database analyses are subject to the limitations of the data recorded. Misclassification of diagnoses and outcomes may occur, only diagnoses coded by the provider will be captured, and any medical service that does not result in the generation of insurance claims (e.g., those paid for out of pocket) will not be captured. The analyses that are the focus in the main text used combined open and closed claims data. Open claims are not yet adjudicated by insurers and can have a greater degree of missing information and patient overlap than closed claims data. Furthermore, this study lacked dental insurance claims data. Most dental/oral surgery procedures will not have been captured in this study due to a lack of inclusion of dental claims in medical insurance claims data for many patients. Additional reasons for lack of dental data include lack of access to or availability of dental services, inability to afford dental care, insurance policies not covering dental care, a lack of education surrounding the importance of dental care, logistical barriers to accessing dental care, or inadequate data capture and reporting even though dental care was given (such as self-pay for dental services not being present in insurance claims databases). A further limitation of claims data is that as physicians and patients become more adept at navigating the claims process for XLH, their claims may increase in a way that does not correlate with disease severity, but rather with increased treatment for stable disease.

Certain important outcomes may be underestimated, such as use of non-opioid pain medications, as these treatments can also be obtained over-the-counter, while phosphate supplements and the active form of vitamin D may not be covered by insurance, thereby not generating an insurance claim and thus not being captured in claims data. Also of note is that while clinicians may test for vitamin D deficiency and treat this with supplementation, they may not code for a deficiency (at all, or on an ongoing basis), lowering the resulting number of claims for vitamin D deficiency in these results. Another limitation relates to vitamin D treatment. When evaluating vitamin D treatment, separating active and non-active vitamin D would have provided a more accurate definition of conventional therapy. Future studies should separate the active and non-active forms of vitamin D to understand the degree and pattern of conventional therapy use in XLH. Further, while the laboratory tests captured here include vitamin D and phosphate levels, the claims analysis did not contain laboratory sampling for other markers, such as parathyroid hormone or bone alkaline phosphatase. This represents a limitation as additional laboratory test data may more clearly define the full burden of XLH.

Additionally, in this study, patients with XLH were identified using diagnosis codes for familial hypophosphatemia and other disorders of phosphorus metabolism since a specific diagnosis code for XLH is not available. In addition, prior to 2016, a code was not available for familial hypophosphatemia specifically. Therefore, non-XLH patients may have been included in this study. The addition of burosumab claims helps minimize the likelihood that we included misdiagnosed patients. However, patients with tumor-induced osteomalacia (TIO), which is also an indication for burosumab in the US, could not be specifically excluded. However, the likelihood of patients with TIO being included is low, as this study required patients be diagnosed with familial hypophosphatemia, and as TIO is extremely rare. In two epidemiology studies the incidence of TIO was below 0.2 per 100,000 person years for the total population of the countries studied [36–38].

In addition, burosumab may occasionally be prescribed off-label for hypophosphatemia due to epidermal nevus syndrome or fibrous dysplasia/McCune-Albright syndrome. However, given that XLH, though rare, is much more common than the other familial hypophosphatemias and renal phosphate wasting disorders, and that burosumab is expected to be only rarely prescribed off-label for the aforenamed conditions, the likelihood that many non-XLH patients were included is expected to be low.

A further limitation is the inclusion of only patients initiating burosumab treatment, which may have skewed the patient population toward those who are more severely affected by XLH or who have greater access to medical therapies. Additionally, the study was unable to identify patients who may have received burosumab therapy during clinical trials prior to the study period, resulting in a potential underestimation of the disease burden due to treatment. However, the impact is expected to be minimal due to the large sample size and the likely small number of patients affected.

The maximum 7-year follow-up of this study likely led to underestimation of the disease burden compared with the cumulative lifetime experience of the disease. For example, events such as procedures may not occur every year but may still be common over the course of many years. Finally, this study did not compare patients who start burosumab with patients who do not. Future research comparing these populations would be of value to understand how trends may change based on burosumab treatment.

## Conclusions

In this large real-world study of patients initiating burosumab for XLH, the majority were aged < 18 years and most were female. The results highlight persistent, and often worsening, trends in morbidities, treatments, and procedures among patients with XLH when stratified by age or examined over time, as well as suggesting that certain complications such as nephrocalcinosis are underdiagnosed. Overall, the study underscores the lifelong impact of XLH and demonstrates the significant burden of disease.

A plain language summary of this article is available in the Online Resources.

## Supplementary Information

Below is the link to the electronic supplementary material.

Supplementary file 1 (PDF 437 KB)

## Data Availability

Data supporting the findings of this study are available from the corresponding author upon reasonable request.
