# Supplementary material for: Real-world characteristics & disease history of patients with X-linked hypophosphatemia before treatment with burosumab
Source: Arch Osteoporos. 2025 May 13;20(1):64. doi: 10.1007/s11657-025-01544-1 (PMC12075324; doi:10.1007/s11657-025-01544-1)
Supplement: Supplementary file 1 — (PDF 437 KB) [file 11657_2025_1544_MOESM1_ESM.pdf]

Real-World Characteristics & Disease History of Patients with X-Linked Hypophosphatemia Before Treatment with Burosumab

ONLINE RESOURCES

Plain Language Summary

- X-linked hypophosphatemia (XLH) is a rare genetic condition that causes low levels of phosphate in the blood. Phosphate is a crucial mineral for strong bones and teeth. Individuals with XLH lack sufficient phosphate, so they tend to have a shorter height, leg deformities, and dental issues such as abscesses
- Due to multiple health problems and trouble doing some activities, people with XLH can have a lower quality of life
- In this study, we aimed to understand the characteristics (e.g., age, sex, geographic location) of people with XLH in the United States, along with their symptoms and the treatments and procedures they received over a 7-year period
- Individuals with XLH in our study experienced numerous symptoms and underwent a wide range of treatments and procedures related to XLH. The most common XLH-related conditions experienced were rickets (70% of patients), joint pain (41%), and leg bowing (39%). The most common XLH-related treatment received was active and non-active forms of vitamin D in 61% of patients
- Symptoms of XLH typically started during childhood, but the percentage of patients experiencing symptoms tended to increase in older age groups. Joint pain, for example, was reported in 12% of those aged 11 years and younger and in 69% of those aged 50 years and over
- This study highlights how XLH affects patients throughout their lives, revealing a heavy burden of the disease

Online Resource Table 1: Patient characteristics, overall and stratified by age group (closed population)

		Index age group				
**Characteristics on index date**	**All Patients N=643**	**≤11 years n=239**	**12–17 years n=137**	**18–29 years n=97**	**30–49 years n=104**	**≥50 years n=66**
**Age, mean ± SD**	21.3 ± 17.6	6.6 ± 2.7	14.3 ± 1.7	21.8 ± 3.4	39.7 ± 5.8	59.4 ± 7.2
**Sex, n (%)**						
Female	396 (61.6)	149 (62.3)	73 (53.3)	66 (68.0)	65 (62.5)	43 (65.2)
**Index year, n (%)**						
2018	74 (11.5)	30 (12.6)	19 (13.9)	7 (7.2)	13 (12.5)	5 (7.6)
2019	261 (40.6)	98 (41.0)	65 (47.4)	40 (41.2)	34 (32.7)	24 (36.4)
2020	144 (22.4)	50 (20.9)	26 (19.0)	28 (28.9)	20 (19.2)	20 (30.3)
2021	114 (17.7)	47 (19.7)	19 (13.9)	11 (11.3)	27 (26.0)	10 (15.2)
2022	50 (7.8)	14 (5.9)	8 (5.8)	11 (11.3)	10 (9.6)	7 (10.6)
**Region, n (%)**						
Northeast	109 (17.0)	36 (15.1)	20 (14.6)	19 (19.6)	16 (15.4)	18 (27.3)
Midwest	174 (27.1)	64 (26. 8)	33 (24.1)	24 (24.7)	28 (26.9)	25 (37.9)
South	279 (43.4)	118 (49.4)	64 (46.7)	37 (38.1)	48 (46.2)	12 (18.2)
West	81 (12.6)	21 (8.8)	20 (14.6)	17 (17.5)	12 (11.5)	11 (16.7)
**Payer channel, n (%)**						
Commercial	348 (54.1)	96 (40.2)	59 (43.1)	62 (63.9)	75 (72.1)	56 (84.8)
Managed Medicaid/Medicaid	283 (44.0)	143 (60.0)	78 (56.9)	34 (35.1)	26 (25.0)	2 (3.0)
Medicare Advantage	12 (1.9)	0 (0.0)	0 (0.0)	1 (1.0)	3 (2.9)	8 (12.1)

Abbreviations: IQR: interquartile range; SD: standard deviation; XLH: X-linked hypophosphatemia

Online Resource Table 2: Patient characteristics in the 5-year subgroup (closed population)

	**All patients**
	**n=56**
**Characteristics on the index date**	
Age, mean ± SD	41.1 ± 16.2
Age group, n (%)	
18–29	19 (33.9)
30–49	17 (30.4)
≥50	20 (35.7)
**Sex, n (%)**	
Female	36 (64.3)
**Index year, n (%)**	
2020	18 (32.1)
2021	25 (44.6)
2022	13 (23.2)
**Region, n (%)**	
Northeast	18 (32.1)
Midwest	14 (25.0)
South	16 (28.6)
West	8 (14.3)
**Payer channel, n (%)**	
Commercial	38 (67.9)
Managed Medicaid/Medicaid	15 (26.8)
Medicare Advantage	3 (5.4)

Online Resource Table 3: Morbidities and other complications in the 5-year subgroup (closed population)

	Year −5<br>n=56	Year −4<br>n=56	Year −3<br>n=56	Year −2<br>n=56	Year −1<br>n=56
**Musculoskeletal manifestations, n (%)**					
Rickets	29 (51.8)	22 (39.3)	19 (33.9)	22 (39.3)	22 (39.3)
Arthralgia	20 (35.7)	23 (41.1)	25 (44.6)	24 (42.9)	21 (37.5)
Genu varum, genu valgum, varus deformities, and coxa vara	7 (12.5)	5 (8.9)	3 (5.4)	3 (5.4)	9 (16.1)
Osteoarthritis	15 (26.8)	13 (23.2)	18 (32.1)	19 (33.9)	20 (35.7)
Difficulty walking	6 (10.7)	2 (3.6)	4 (7.1)	5 (8.9)	6 (10.7)
Short stature	5 (8.9)	2 (3.6)	1 (1.8)	1 (1.8)	4 (7.1)
Fracture	9 (16.1)	7 (12.5)	7 (12.5)	8 (14.3)	8 (14.3)
Muscle weakness	3 (5.4)	3 (5.4)	1 (1.8)	2 (3.6)	4 (7.1)
Enthesopathy	4 (7.1)	6 (10.7)	4 (7.1)	6 (10.7)	7 (12.5)
Myalgia	1 (1.8)	6 (10.7)	4 (7.1)	5 (8.9)	6 (10.7)
Spinal stenosis	0 (0.0)	1 (1.8)	4 (7.1)	2 (3.6)	3 (5.4)
Osteomalacia	3 (5.4)	2 (3.6)	5 (8.9)	6 (10.7)	7 (12.5)
Delayed growth/delayed walking	0 (0.0)	0 (0.0)	0 (0.0)	0 (0.0)	0 (0.0)
Craniosynostosis	1 (1.8)	0 (0.0)	0 (0.0)	1 (1.8)	0 (0.0)
Chiari malformation	0 (0.0)	0 (0.0)	1 (1.8)	0 (0.0)	0 (0.0)
**Other symptoms and conditions of interest, n (%)**					
Obesity	16 (28.6)	17 (30.4)	20 (35.7)	18 (32.1)	19 (33.9)
Vitamin D deficiency	13 (23.2)	12 (21.4)	16 (28.6)	19 (33.9)	16 (28.6)
Renal disease	9 (16.1)	10 (17.9)	11 (19.6)	9 (16.1)	12 (21.4)

	Year −5<br><br>n=56	Year −4<br><br>n=56	Year −3<br><br>n=56	Year −2<br><br>n=56	Year −1<br><br>n=56
Hypertension	18 (32.1)	19 (33.9)	23 (41.1)	26 (46.4)	25 (44.6)
Depression	9 (16.1)	6 (10.7)	7 (12.5)	10 (17.9)	11 (19.6)
Hearing loss	2 (3.6)	0 (0.0)	1 (1.8)	7 (12.5)	6 (10.7)
Nephrocalcinosis	1 (1.8)	3 (5.4)	2 (3.6)	2 (3.6)	2 (3.6)
Hyperparathyroidism	7 (12.5)	5 (8.9)	5 (8.9)	6 (10.7)	7 (12.5)
Kidney stone	3 (5.4)	6 (10.7)	4 (7.1)	5 (8.9)	4 (7.1)

Online Resource Figure 1: Included patient population (closed population)

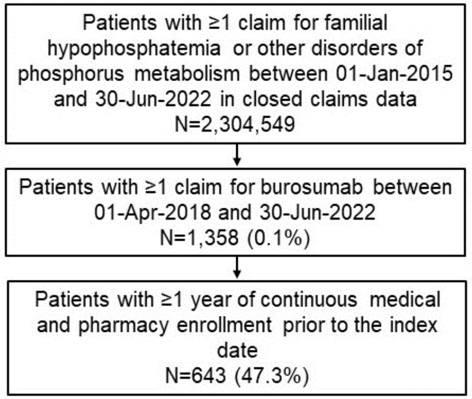

Online Resource Figure 2a: Musculoskeletal manifestations over the 1-year pre-index period, by age group (closed population)

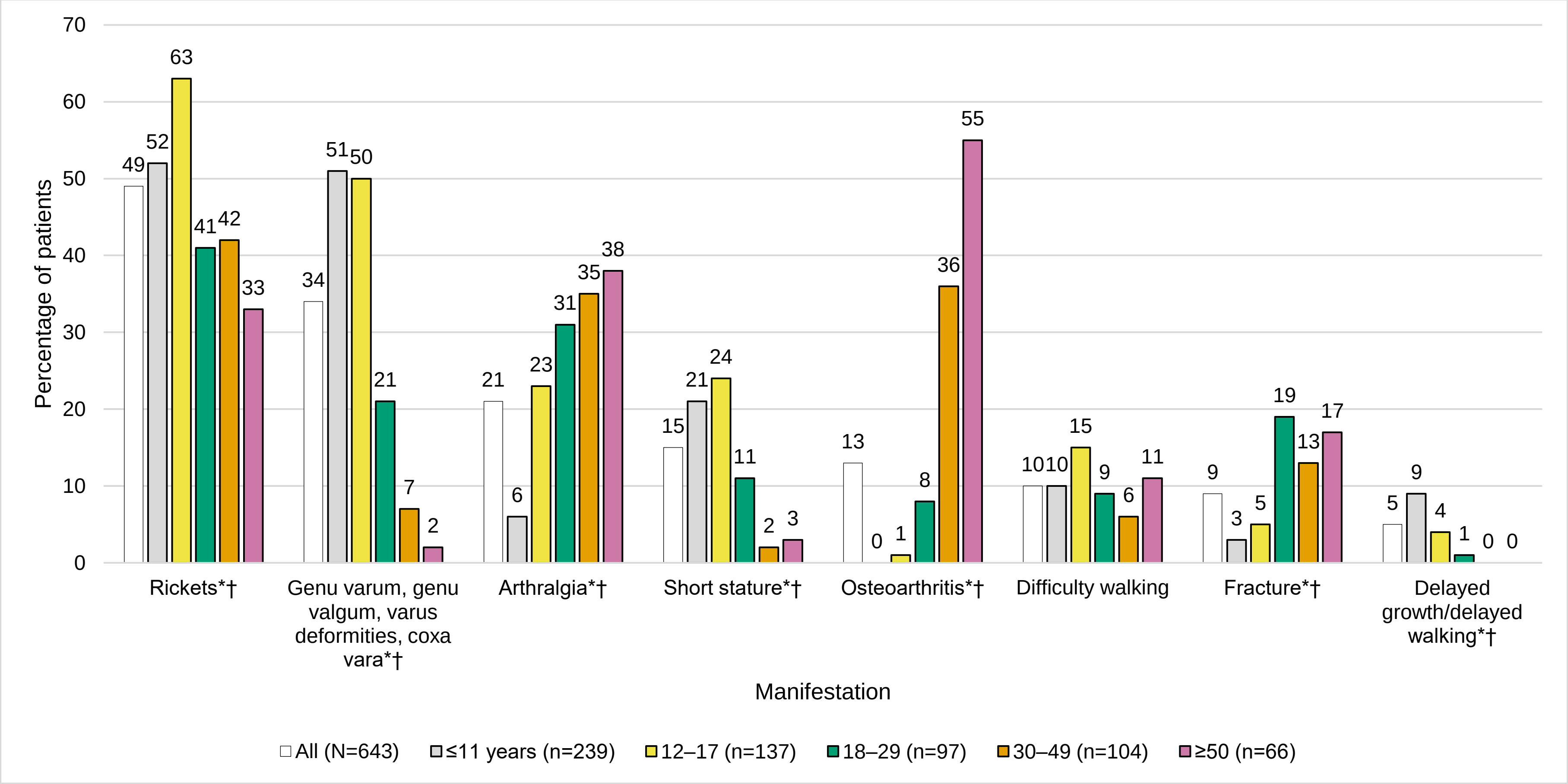

*P<0.01 for differences in proportions.
†P<0.01 for trend in proportions.

For these outcomes stratified by age group, the chi-square test (or Fisher exact test if any count was <5) was used to test differences in the distribution of morbidities, other complications, and treatments in the 1-year prior to index among the age groups, and the Cochran-Armitage test was used to assess the presence of any associations.

Fig 2a. The bar chart depicts musculoskeletal manifestations determined by diagnosis coding, by age group in the 1-year pre-index period in the closed population. The Y-axis is the percentage of patients, and the X-axis is manifestations, with each manifestation having a bar for the overall population and a separate bar for each age category. The number of patients in each category is: overall 643; ≤11 years 239; 12–17 years 137; 18–29 years 97; 30–49 years 104; and ≥50 years 66. The percentage of patients with each manifestation, listed in the order they appear from left to right (starting with the overall population, and continuing from youngest to oldest age group) is: rickets (49, 52, 63, 41, 42, 33); genu varum, genu valgum, varus deformities, and coxa vara (34, 51, 50, 21, 7, 2); arthralgia (21, 6, 23, 31, 35, 38); short stature (15, 21, 24, 11, 2, 3); osteoarthritis (13, 0, 1, 8, 36, 55); difficulty walking (10, 10, 15, 9, 6, 11); fracture (9, 3, 5, 19, 13, 17); delayed growth/delaying walking (5, 9, 4, 1, 0, 0). All manifestations except difficulty walking have P<0.01 for differences in proportions and P<0.01 for trend in proportions.

Online Resource Figure 2b: Additional musculoskeletal manifestations over the 1-year pre-index period, by age group (closed population)

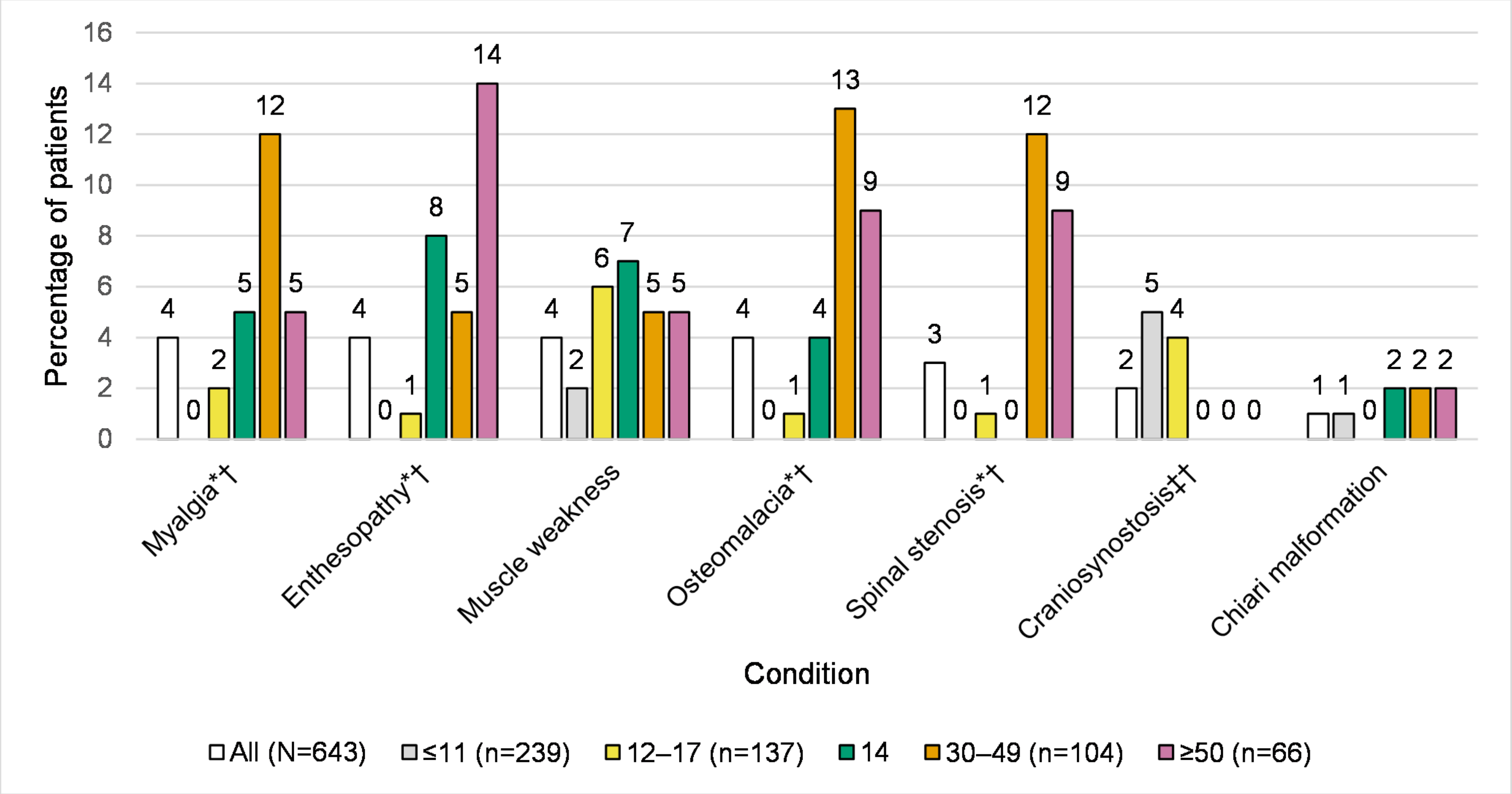

*P<0.01 for differences in proportions.
†P<0.01 for trend in proportions.
‡P<0.05 for differences in proportions.

For these outcomes stratified by age group, the chi-square test (or Fisher exact test if any count was <5) was used to test differences in the distribution of morbidities, other complications, and treatments in the 1-year prior to index among the age groups, and the Cochran-Armitage test was used to assess the presence of any associations.

Fig 2b. The bar chart depicts additional musculoskeletal manifestations determined by diagnosis coding, by age group in the 1-year pre-index period in the closed population. The Y-axis is the percentage of patients, and the X-axis is manifestation, with each manifestation having a bar for the overall population and a separate bar for each age category. The number of patients in each category is: overall 643; ≤11 years 239; 12–17 years 137; 18–29 years 97; 30–49 years 104; and ≥50 years 66. The percentage of patients with each manifestation, listed in the order they appear from left to right (starting with the overall population, and continuing from youngest to oldest age group) is: myalgia (4, 0, 2, 5, 12, 5); enthesopathy (4, 0, 1, 8, 5, 14); muscle weakness (4, 2, 6, 7, 5, 5); osteomalacia (4, 0, 1, 4, 13, 9); spinal stenosis (3, 0, 1, 0, 12, 9); craniosynostosis (2, 5, 4, 0, 0, 0); chiari malformation (1, 1, 0, 2, 2, 2). The following manifestations have $P<0.01$ for differences in proportions: myalgia, enthesopathy, osteomalacia, spinal stenosis. The following manifestations have $P<0.01$ for trend in proportions: myalgia, enthesopathy, osteomalacia, spinal stenosis, craniosynostosis. Craniosynostosis is the only manifestation with $P<0.05$ for differences in proportions.

Online Resource Figure 3: Other symptoms and conditions of interest over the 1-year pre-index period, by age group (closed population)

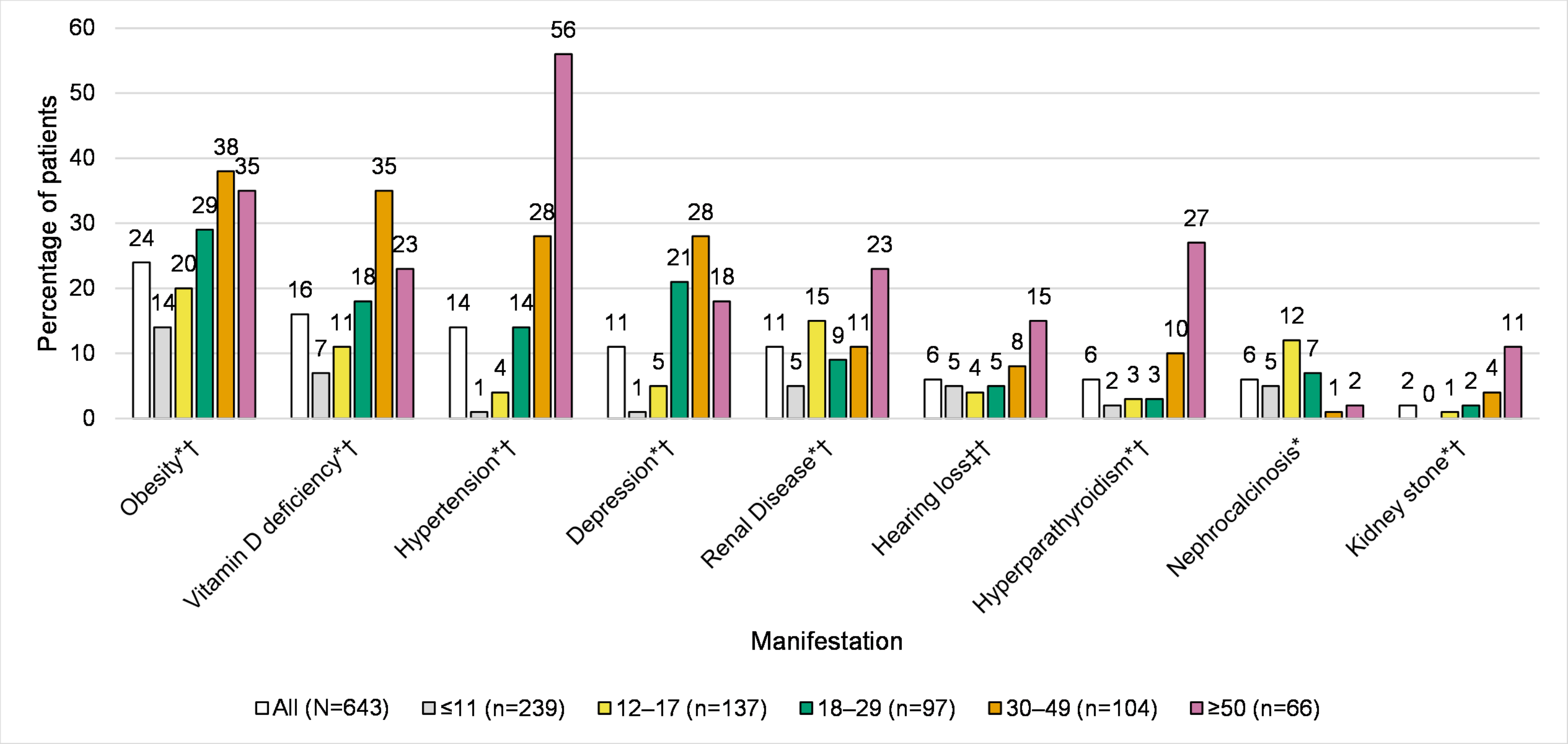

*P<0.01 for differences in proportions.
†P<0.01 for trend in proportions.
‡P<0.05 for differences in proportions.

For these outcomes stratified by age group, the chi-square test (or Fisher exact test if any count was <5) was used to test differences in the distribution of morbidities, other complications, and treatments in the 1-year prior to index among the age groups, and the Cochran-Armitage test was used to assess the presence of any associations.

Fig 3. The bar chart depicts other symptoms and conditions of interest determined by diagnosis coding, by age group in the 1-year pre-index period in the closed population. The Y-axis is the percentage of patients, and the X-axis is symptom or condition, with each symptom or condition having a bar for the overall population and a separate bar for each age category. The number of

patients in each category is: overall 643; ≤11 years 239; 12–17 years 137; 18–29 years 97; 30–49 years 104; and ≥50 years 66. The percentage of patients with each symptom or condition of interest, listed in the order they appear from left to right (starting with the overall population, and continuing from youngest to oldest age group) is: obesity (24, 14, 20, 29, 38, 35); vitamin D deficiency (16, 7, 11, 18, 35, 23); hypertension (14, 1, 4, 14, 28, 56); depression (11, 1, 5, 21, 28, 18); renal disease (11, 5, 15, 9, 11, 23); hearing loss (6, 5, 4, 5, 8, 15); hyperparathyroidism (6, 2, 3, 3, 10, 27); nephrocalcinosis (6, 5, 12, 7, 1, 2); kidney stone (2, 0, 1, 2, 4, 11). The following symptoms and conditions have $P<0.01$ for differences in proportions: obesity, vitamin D deficiency, hypertension, depression, renal disease, hyperparathyroidism, nephrocalcinosis, kidney stone. The following symptoms and conditions have $P<0.01$ for trend in proportions: obesity, vitamin D deficiency, hypertension, depression, renal disease, hearing loss, hyperparathyroidism, kidney stone. Hearing loss was the only symptom or condition with $P<0.05$ for differences in proportions.

Online Resource Figure 4a: Medical treatments over the 1-year pre-index period, by age group (closed population)

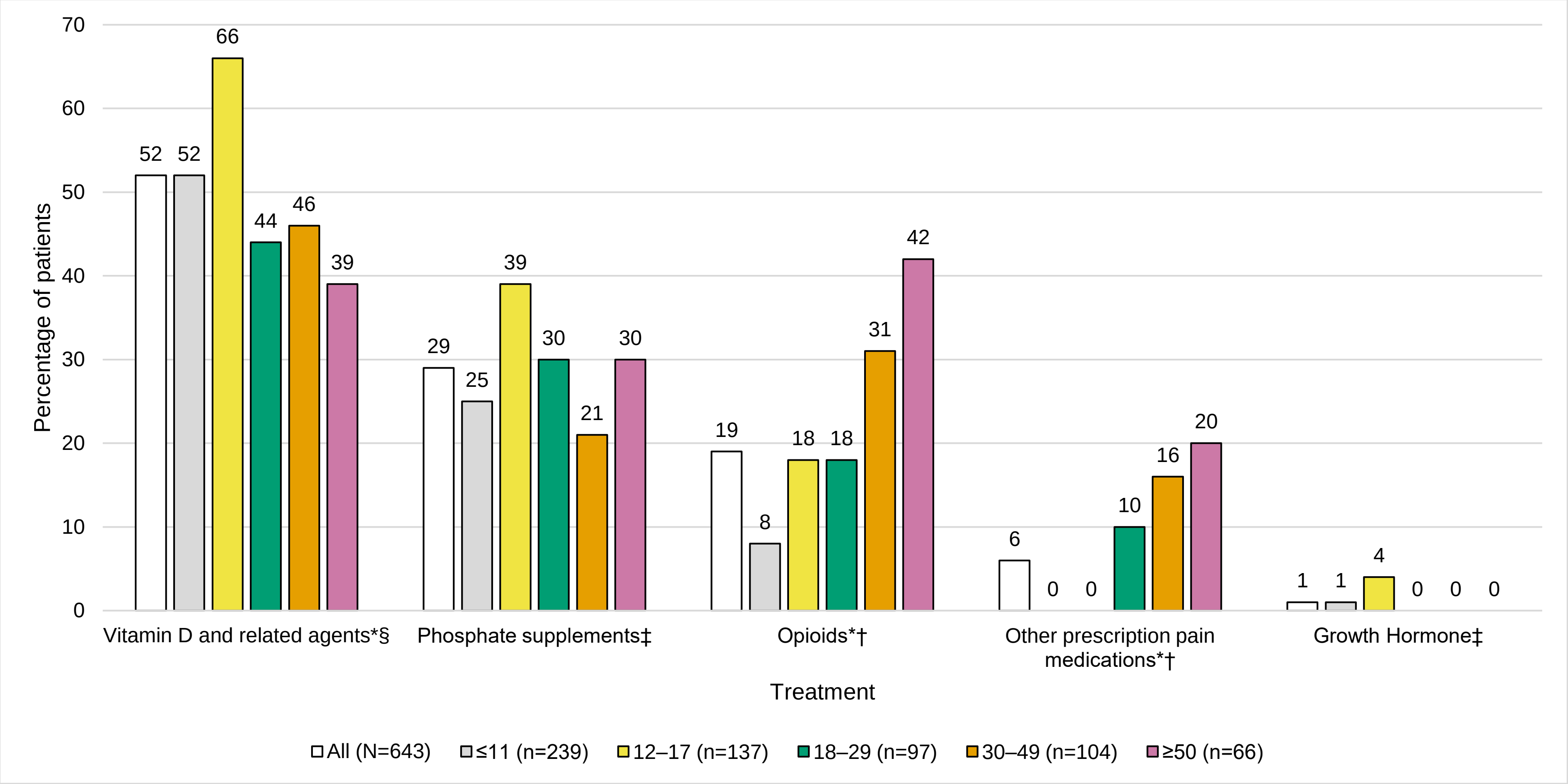

*P<0.01 for differences in proportions.
†P<0.01 for trend in proportions.
‡P<0.05 for differences in proportions.
§P<0.05 for trend in proportions.

For these outcomes stratified by age group, the chi-square test (or Fisher exact test if any count was <5) was used to test differences in the distribution of morbidities, other complications, and treatments in the 1-year prior to index among the age groups, and the Cochran-Armitage test was used to assess the presence of any associations.

Fig 4a. The bar chart depicts medical treatments determined by diagnosis coding, by age group in the 1-year pre-index period in the closed population. The Y-axis is the percentage of patients, and the X-axis is treatment, with each treatment having a bar for the overall population and a separate bar for each age category. The number of patients in each category is: overall 643; ≤11 years 239; 12–17 years 137; 18–29 years 97; 30–49 years 104; and ≥50 years 66. The percentage of patients with each treatment, listed in the order they appear from left to right (starting with the overall population, and continuing from youngest to oldest age group) is: vitamin D and related agents (52, 52, 66, 44, 46, 39); phosphate supplements (29, 25, 39, 30, 21, 30); opioids (19, 8, 18, 18, 31, 42); other prescription pain medications (6, 0, 0, 10, 16, 20); growth hormone (1, 1, 4, 0, 0, 0). The following treatments have P<0.01 for trend in proportions: vitamin D and related agents, opioids, other prescription pain medications. The following treatments have P<0.01 for trend in proportions: opioids, other prescription pain medications. The following treatments have P<0.05 for differences in proportions: phosphate supplements, growth hormone. Vitamin D and related agents was the only treatment with P<0.05 for trend in proportions.

Online Resource Figure 4b: Other treatments and procedures over the 1-year pre-index period, by age group (closed population)

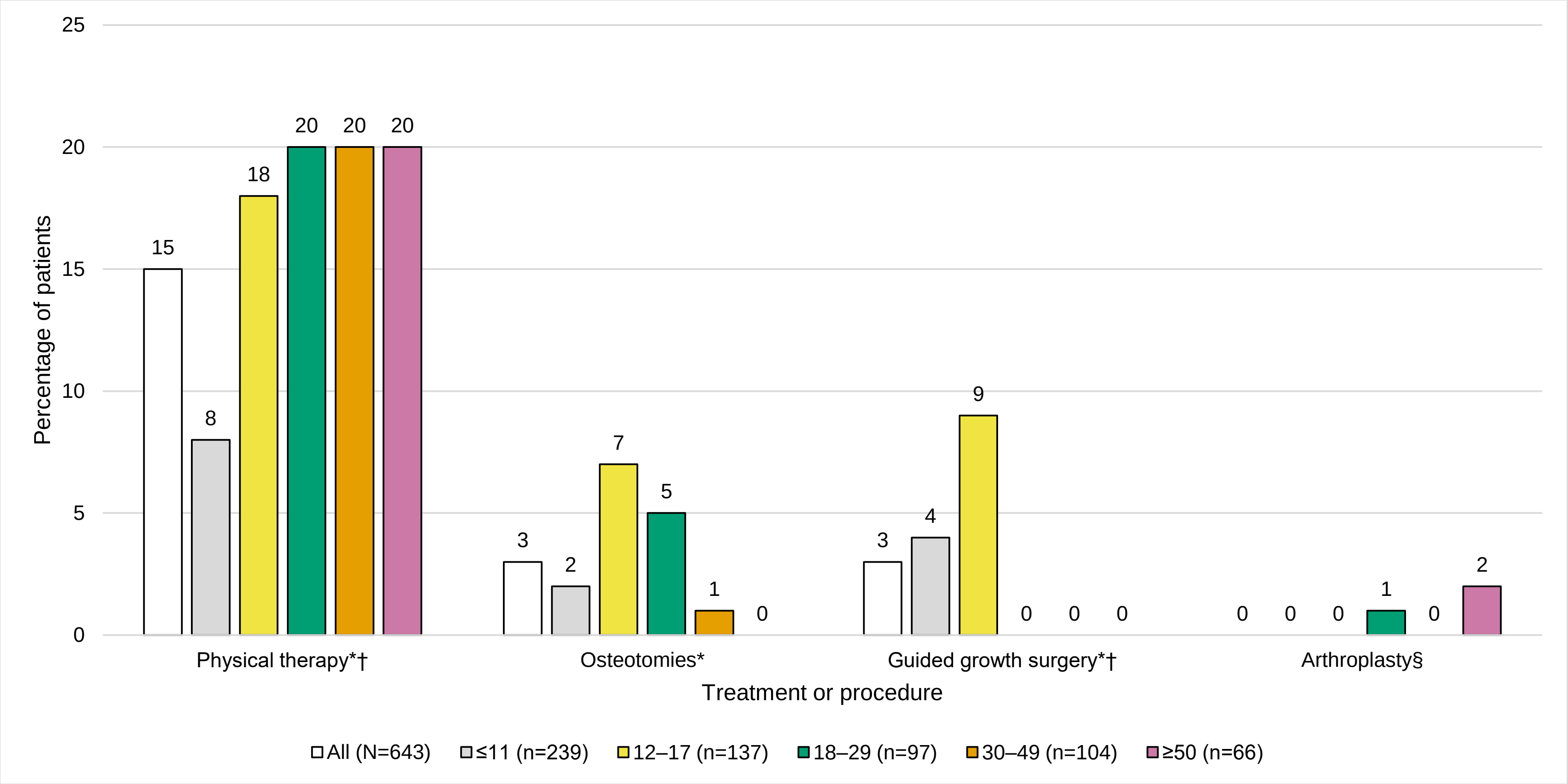

*P<0.01 for differences in proportions.
†P<0.01 for trend in proportions.
§P<0.05 for trend in proportions.

For these outcomes stratified by age group, the chi-square test (or Fisher exact test if any count was <5) was used to test differences in the distribution of morbidities, other complications, and treatments in the 1-year prior to index among the age groups, and the Cochran-Armitage test was used to assess the presence of any associations.

Fig 4b. The bar chart depicts other treatments and procedures by diagnosis coding, by age group in the 1-year pre-index period in the closed population. The Y-axis is the percentage of patients, and the X-axis is treatment or procedure, with each treatment or procedure having a bar for the overall population and a separate bar for each age category. The number of patients in each category is: overall 643; ≤11 years 239; 12–17 years 137; 18–29 years 97; 30–49 years 104; and ≥50 years 66. The percentage of patients with each treatment or procedure, listed in the order they appear from left to right (starting with the overall population, and continuing from youngest to oldest age group) is: physical therapy (15, 8, 18, 20, 20, 20); osteotomies (3, 2, 7, 5, 1, 0); guided growth surgery (3, 4, 9, 0, 0, 0); arthroplasty (0, 0, 0, 1, 0, 2). All treatments and procedures except arthroplasty have P<0.01 for differences in proportions. Physical therapy and guided growth surgery have P<0.01 for trend in proportions. Arthroplasty was the only treatment or procedure with P<0.05 for trend in proportions.
